# Surgical Training and Education in Promoting Professionalism: a comparative assessment of virtue-based leadership development in otolaryngology-head and neck surgery residents

**DOI:** 10.3402/meo.v18i0.22440

**Published:** 2013-10-29

**Authors:** Kristine Schulz, Liana Puscas, Debara Tucci, Charles Woodard, David Witsell, Ramon M. Esclamado, Walter T. Lee

**Affiliations:** 1 Division of Otolaryngology, Head and Neck Surgery, Duke University Medical Center, Durham, NC, USA; 2 Section of Otolaryngology, Head and Neck Surgery, Durham VA Medical Center, Durham, NC, USA

**Keywords:** professionalism, residency, virtues, ethics, character training

## Abstract

**Introduction:**

Surgical Training and Education in Promoting Professionalism (STEPP) was developed in 2011 to train tomorrow's leaders during residency. It is based on virtue ethics and takes an approach similar to West Point military academy. The purpose of this research was: (i) to compare the virtue profiles of our residents with that of the military cohort using a standardized virtue assessment tool; and (ii) to assess the value of virtue education on residents.

**Methods:**

As part of STEPP, otolaryngology residents participated in a virtue-based validated assessment tool called Virtue in Action (VIA) Inventory. This was completed at the initiation of STEPP in July 2011 as well as 1 year later in June 2012. Comparison of the VIA to a military cohort was performed. Leadership ‘Basic Training’ is a series of forums focused on virtues of initiative, integrity, responsibility, self-discipline, and accountability. A pre- and post-test was administered assessing resident perceptions of the value of this ‘Basic Training’.

**Results:**

Virtues are shared between otolaryngology residents (*n*=9) and military personnel (*n*=2,433) as there were no significant differences in strength scores between two military comparison groups and otolaryngology-head and neck surgery (OHNS) residents. There was a significant improvement (*p*<0.001) in the understanding of components of the leadership vision and a significant improvement in the understanding of key leadership concepts based on ‘Basic Training’. All residents responded in the post-test that the STEPP program was valuable, up from 56%.

**Conclusions:**

A virtue-based approach is valued by residents as a part of leadership training during residency.

## Introduction

It is within West Point's mission to emphasize honor and integrity. The West Point mission reads: ‘To educate, train, and inspire the Corps of Cadets so that each graduate is a commissioned leader of character committed to the values of Duty, Honor, Country and prepared for a career of professional excellence and service to the Nation as an officer in the United States Army’ (1). Offstein and Dufresne (2) explored the design and process of ethical and character development at the US Military Academy at West Point. Based on a combined approach using interviews and archived data, the authors conclude that the character-development approach utilized at West Point – one that infuses all levels from recruiting and selection, mission statements and honor codes, and job rotations – can be a model for non-military settings for attaining higher standards of ethics.

The parallels between military and medicine support the idea that similar approaches may be translatable to the healthcare setting. The military is reliant on its ability to quickly form effective teams (3). This tenet is crucial in medicine as well and the inability to do so has implications on safety, wellbeing, and the greater good (4). Furthermore, as pointed out by Cycyota et al. (5) and Seiler et al. (6), the development of skills to support moral decision making and implementing processes for ethical development of leaders is imperative to developing leaders that embody respect for human dignity and are culturally competent. Again, these same ideals contribute directly to the interaction, decision making, and care of patients and their families.

Attention to the importance of the development of leaders and effective teams in medicine is not new; however, much of the literature focuses on process and remedy through communication techniques or systems science approaches. The importance of effective communication, teamwork, and respect across team members in medical settings has been well documented along with the impact and proposed strategies (4, 7, 8). Strategies, such as using applied organizational theories and systems science to improve relationships have also yielded potential models for the development of effective leaders and teams (9). While these approaches have merit and can be effective, they focus on methods and strategies – physicians after all are scientists – and therefore this process of assessing or diagnosing and applying a treatment or remedy ‘to fix the problem’ is not surprising yet sustainable change is difficult.

Recently, medicine has seen a shift toward the mindset long held by military – effective teams and effective leaders are achieved through the individual development of each person's character. The paradigm is shifting in medicine to a more comprehensive view of the patient. Much in the way that a better overall tending to one's health and health status yields fewer aches and pains and comorbidities, the overall tending to one's character could allow us to self-evolve into leaders of character. Through individual assessment of one's strengths and weaknesses, we have begun to understand that the creation of leaders of character in medicine hinges upon understanding more about ourselves (10). We have begun a journey in medicine to rethink the litmus for what indicates leadership success. Souba (11) describes the need for a realigned leadership framework, one that is based on leadership character pillars of awareness, commitment, integrity, and authenticity. In medicine, we need to develop leaders of character who embody the Hippocratic oath the way the cadets and future military leaders from West Point embody their mission.

Based upon this emerging need in medicine for contemporary leadership, our residency program at Duke has sought to develop a training curriculum in inform residents about leadership principles. Our educational mission in the Division of Otolaryngology-Head and Neck Surgery is ‘to train tomorrow's leaders’. This cannot happen passively. Therefore, we developed and implemented a leadership curriculum to the residents through a modular format. Herein, we describe our program and begin to examine its effectiveness compared to a cohort of West Point Cadets.

Duke University's Division of Otolaryngology-Head and Neck Surgery (OHNS) faculty identified five core leadership qualities to incorporate in resident education: initiative; integrity; self-discipline; responsibility; and accountability. Teaching professionalism in medicine is often done through implicit ‘leading by example’ with little direct programming within their clinical experience to give residents tools to utilize in their leadership growth. Duke OHNS believes that both approaches are integral to this initiative.

## Methods

During the summer of 2011, a pilot program for a professionalism curriculum was developed for faculty and residents within Duke OHNS under the leadership of Walter Lee, MD. Called *STEPP* – ***S***urgical ***T***raining and ***E***ducation ***P***romoting ***P***rofessionalism, the program aims to provide a foundation and pathway, or steps, for becoming tomorrow's leaders.

STEPP's vision is ‘to train tomorrow's leaders’ (inclusive of everyone regardless of status and position) and the program's aims are to:

1. Provide proactive and explicit leadership training;
2. Establish a basic and common foundational understanding of the five identified core leadership qualities;
3. Provide examples and opportunities for application of these five qualities in Duke OHNS residency training.

The key components of the program are:

1. Virtue Strength Assessments (Virtue in Action [VIA] tool): This extensive online survey through VIA Institute on Character (www.viacharacter.org) gives individualized feedback to participants on their character strengths, from which to reflect during the course of the program. Initial assessments for the pilot were performed at the start of the program in September 2011. There is precedence in the literature for utilizing the VIA tool to identify character strengths that are indicative of achievement in leadership roles (http://viacharacter.org/www/en-us/research/summaries.aspx#achievement). The six overarching categories of character strengths along with their underlying component areas are:
  - Wisdom and knowledge (creativity, curiosity, judgment/open-mindedness, love of learning, perspective);
  - Courage (bravery, perseverance, honesty, zest);
  - Humanity (love, kindness, social intelligence);
  - Justice (teamwork, fairness, leadership);
  - Temperance (forgiveness/mercy, modesty/humility, prudence, self-regulation);
  - Transcendence (appreciation of beauty/excellence, gratitude, hope, humor, religiousness/spirituality).
2. Mentorship meetings among faculty and staff.
3. ‘Thought of the Day’ to Division's faculty and staff (e.g., ‘Never sacrifice fundamental beliefs or core values as you adjust to ever-changing external factors’.)
4. Internal and external faculty training and development (Faculty participation in both internally available Duke programs in leadership and professionalism along with external opportunities).
5. Leadership basic training course for residents (interactive sessions mixed with required pertinent readings) developed and taught in monthly sessions by OHNS faculty.

### Interim evaluation of STEPP for resident leadership development

Our long-term hypothesis is that the STEPP program will result in gains by the OHNS residents in key character strengths and provide the foundation for training tomorrow's leaders. This interim analysis provides a state of the union and baseline data on the first 6 months of the STEPP program.

In January 2012, an interim analysis was performed utilizing the data available to-date on the nine OHNS residents in the program.

1. Pre-/post-test comparison.
2. Correlation analysis between post-test assessment of self-perceived strengths versus VIA strength profile.
3. Comparison of character strength average scores of OHNS residents to benchmark groups from a military cohort from the VIA tool database and West Point Cadets from publications using the VIA tool.

The Duke University Health System Institutional Review Board (IRB) declared the study exempt.

## Results

### Pre-/post-test comparison

**Fig. 1:**
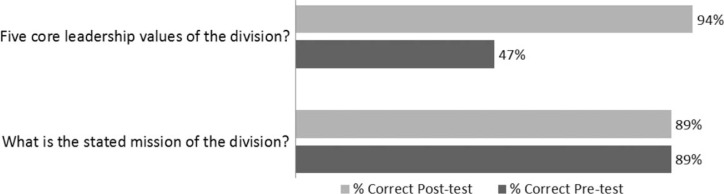
Percent of residents correctly answering questions regarding core leadership values and stated mission.

**Fig. 2:**
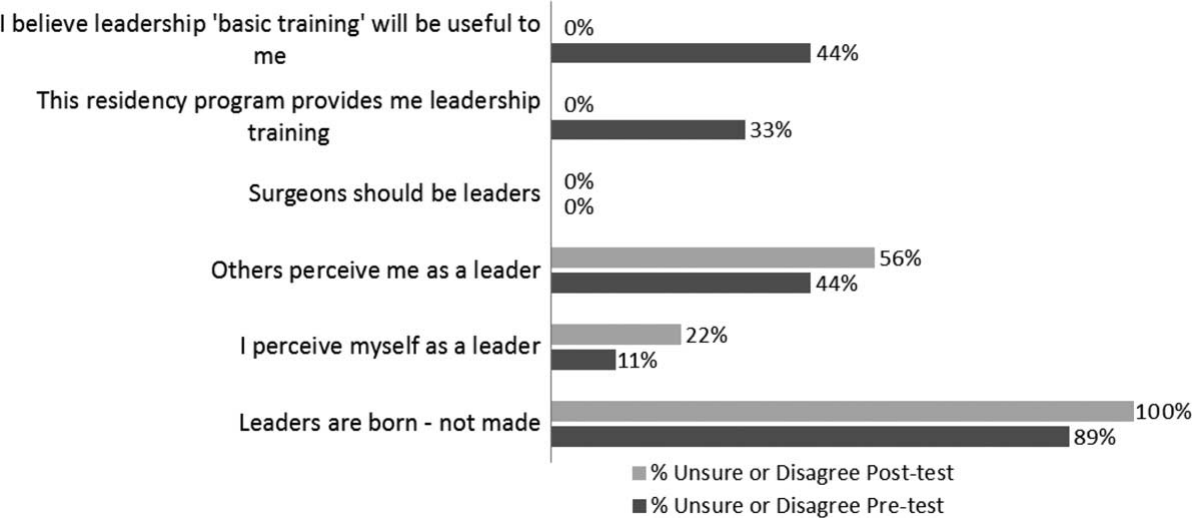
Percent of residents unsure or disagree with leadership-related belief statements.

A pre-test was administered at the start of the program in August 2011 and a post-test was performed in January 2012. The results confirmed that there is a need for and opportunities to enhance the leadership and professionalism training for our residents and future leaders. The survey found that close to 50% of the respondents did not think or were unsure of whether others perceived them as leaders. The survey also found that one-third of the respondents were unsure of whether their residency experience provides them with leadership training and over half were unsure as to whether leadership training will be helpful to them in the future. Using a paired *t*-test (*p*<0.001), there was a significant improvement in the residents’ understanding of the OHNS Division values, as evidenced by being able to list the five core values – on the pre-test, only 47% of the responses to the open-ended question to list the five core values were correct versus 94% on the post-test (mean difference (95% CI) 46.3 (69.0, 23.5) SD (95% CI) 27.2 (18.0, 55.4)). On the post-test, 100% of the residents felt the program would be beneficial to them, up from 56% on the pre-test. Figures 1 and 2 present the results of the pre- and post-tests.

### Correlation analysis

The nonparametric Pearson rank test was used to test association strength between pre-test assessment of self-perceived strengths versus VIA strength profile. A correlation was performed on resident response to the question ‘Others perceive me as a leader’ and their VIA character score for leadership. Missing data for one pre-test only allowed for a comparison of eight resident's data. There is a very strong correlation (0.85) between VIA score (0–5 with 5 as the highest) for the character strength of leadership and the direct self-assessment on the pre-test that ‘others perceive me as a leader’ (0–2; 0 = No; 1 = Unsure; 2 = Yes). There is also a strong correlation (0.55) between VIA score for the character strength of leadership and whether the resident felt ‘leadership basic training would be useful to me’ (0–2; 0 = No; 1 = Unsure; 2 = Yes).

### Comparison of OHNS residents to benchmark groups

Table 1 presents the results of the comparison of OHNS resident character scores to a benchmark military group (n = 2,433) from the VIA survey database. Two of the five top-ranked character strengths match – ‘honesty’ and ‘judgment and open-mindedness’. The other three top rankings in the resident group are ‘curiosity’ which ranks first in the resident group and ninth in the military group, ‘capacity to love or be loved’ which ranks fourth in the resident group and 11th in the military group, and finally ‘creativity’ which ranks fifth in the resident group and 16th in military group. The other three top rankings in the military group are ‘fairness’ which ranks second in the military group and eighth in the resident group; ‘kindness’ which ranks fourth in the military group and ninth in the resident group; and ‘perseverance’ which ranks fifth in the military group and 11th in the resident group. Table 2 presents the character strengths rolled up into the six virtue categories where both the resident and military groups have ranks of 2 for ‘courage’ and 3 for ‘humanity’ and differ on ‘justice’ (rank 1 military vs. 5 residents); ‘temperance’ (rank 4 military vs. 6 residents); ‘transcendence’ (rank 5 military vs. 4 residents); and ‘wisdom & knowledge’ (rank 6 military vs. 1 residents). When compared using a two-tailed *t*-statistic, there are no character strength or virtue category scores that differ with statistical significance at *p*<0.05. Using data from a study by Matthews et al. (12), Table 3 compares the resident data by virtue category to that of West Point Cadets. Again, there are no statistically significant differences in scores between the two groups. In terms of ranking, both the residents and cadets have ranks of 2 for ‘humanity’ and 6 for ‘temperance’ and differ on ‘courage’ (rank 1 cadets vs. 3 residents); ‘justice’ (rank 3 cadets vs. 5 residents); ‘transcendence’ (rank 5 for cadets vs. 4 residents); and ‘wisdom and knowledge’ (rank 4 cadets vs. 1 residents).

**Table 1:** Comparison of OHNS residents to VIA database ‘Military’ surveys

		Military (military respondents in VIA database; *n*=2,433)			OHNS residents (n = 9)				
				
Virtue category	Character strength	Mean	SD	Rank	Mean	SD	Rank	% ± military	p*
Courage	Bravery	3.977	0.568	8	3.733	0.505	*20*	−6.5	0.199
	Honesty	4.148	0.504	1	4.189	0.341	2	1.0	0.807
	Perseverance	4.008	0.581	5	3.867	0.245	11	−3.6	0.467
	Zest	3.762	0.619	17	3.800	0.377	15	1.0	0.854
Humanity	Kindness	4.023	0.562	4	3.911	0.504	9	−2.9	0.551
	Capacity to love and be loved	3.943	0.595	11	4.067	0.328	4	3.0	0.532
	Social intelligence	3.890	0.571	15	3.789	0.376	16	−2.7	0.596
Justice	Fairness	4.043	0.556	2	3.967	0.648	8	−1.9	0.683
	Leadership	3.893	0.560	14	3.622	0.570	*23*	−7.5	0.148
	Teamwork	3.943	0.553	12	3.822	0.661	13	−3.2	0.513
Temperance	Forgiveness and mercy	3.614	0.723	*21*	3.700	0.477	*21*	2.3	0.721
	Modesty and humility	3.598	0.647	*22*	3.467	0.527	*24*	−3.8	0.544
	Prudence	3.565	0.628	*23*	3.689	0.333	*22*	3.4	0.554
	Self-regulation	3.723	0.606	19	3.778	0.360	17	1.5	0.786
Transcendence	Appreciation of beauty and excellence	3.449	0.762	*24*	3.767	0.857	19	8.4	0.212
	Gratitude	3.963	0.616	10	3.856	0.410	12	−2.8	0.603
	Hope	3.991	0.613	7	3.978	0.479	7	−0.3	0.949
	Humor	3.992	0.614	6	3.878	0.482	10	−2.9	0.578
	Religiousness and spirituality	3.737	0.887	18	3.989	0.513	6	6.3	0.395
Wisdom and knowledge	Creativity	3.821	0.644	16	4.056	0.403	5	5.8	0.274
	Curiosity	3.973	0.544	9	4.322	0.589	1	8.1	0.055
	Judgment and open-mindedness	4.026	0.537	3	4.156	0.482	3	3.1	0.469
	Love of learning	3.644	0.669	*20*	3.778	0.466	18	3.5	0.549
	Perspective	3.923	0.544	13	3.811	0.379	14	−2.9	0.537

* Two-tailed *t*-test.

OHNS, otolaryngology, head and neck surgery; VIA, Virtue in Action.

**Table 2:** Comparison of OHNS residents to VIA database ‘Military’ surveys (category level)

	Military (*n*=2,433)			OHNS residents (*n*=9)			
			
Virtue category	Mean	SD	Rank	Mean	SD	Rank	p*
Courage	3.947	0.568	3	3.897	0.403	3	0.792
Humanity	3.952	0.576	2	3.922	0.410	2	0.876
Justice	3.960	0.556	1	3.804	0.620	5	0.401
Temperance	3.625	0.651	4	3.658	0.429	6	0.879
Transcendence	3.826	0.699	5	3.893	0.550	4	0.774
Wisdom and knowledge	3.877	0.588	6	4.024	0.494	1	0.454

* Two-tailed *t*-test.

OHNS, otolaryngology, head and neck surgery; VIA, Virtue in Action.

**Table 3:** Comparison of OHNS residents to West Point Cadets from Matthew et al. (2006)

	Matthews et al. (2006)						
		
	West Point Cadets (*n*=103; mean age 18.3)			OHNS residents (*n*=9; mean age 30.8)			
			
Virtue category	Mean	SD	Rank	Mean	SD	Rank	p*
Courage	4.010	0.390	1	3.897	0.403	3	0.408
Humanity	3.980	0.470	2	3.922	0.410	2	0.721
Justice	3.930	0.490	3	3.804	0.620	5	0.471
Temperance	3.710	0.440	6	3.658	0.429	6	0.734
Transcendence	3.870	0.500	5	3.893	0.550	4	0.896
Wisdom and knowledge	3.890	0.440	4	4.024	0.494	1	0.387

* Two-tailed *t-*test.

OHNS, otolaryngology, head and neck surgery.

Permission received from Michael D. Matthews.

## Discussion

The interim analysis indicates that there is a need for and positive response to leadership and professionalism training in residency. Professionalism training is not at the forefront of a resident's thoughts each day – their clinical and research training takes precedence. Improvement in their recitation of the OHNS division values may indicate that the program is at the minimum accepted and ideally has begun infusing the values in a way that they become part of the culture and embodied in their character. With the program only at the 6-month mark, it is too soon to make major assumptions based on pre- and post-test data. The increase to 100% consensus that the program will be beneficial is also a key marker. Of interest is the correlation between the pre-test response to the question on the perceived usefulness of the program and VIA character score. The correlation may indicate that those that already embody strong leadership character strengths have a better innate understanding of the value of lessons in character development.

If we agree even partially with the assumption that the military as a whole more comprehensively embodies the oath they take more so than physicians as a whole in modern-day medicine, we can potentially learn from both their approach to training and their profile of strengths. The comparison of the resident data to that of the military group and a perhaps more reflective peer comparison in West Point Cadets, shows no overall virtue categories or their comprising character strengths with statistically different scores. This lends some credence to confirming that the types of men and women that pursue these careers have similar profiles with variations in areas we would expect with the two strengths closest to significant differences being higher ‘curiosity’ and ‘appreciation of beauty and excellence’ scores in the resident group and higher ‘bravery’ and ‘leadership’ scores in the military group. While some differences may make sense, there are others upon which we can reflect as potential areas of exploration and growth. For example, the top-ranked virtue category based on the military group scores was ‘justice’, which is a rank of 5 in the resident group. Within ‘justice’, the comprising categories of ‘fairness’, ‘leadership’, and ‘teamwork’, while not statistically significantly different, each had scores that were lower than the military group. These character traits are important to our endeavor ‘to train tomorrow's leaders’ through our STEPP program with residents, and therefore may be areas upon which to focus in training and additionally, to learn more from our military counterparts in terms of training and educational approaches. There is no right or wrong to character strengths and one cannot assume that a given set of strengths will guarantee or impede success. It is however interesting in this journey to shift the paradigm in medicine and focus on character-based role models, such as the military, to use for introspection and as a frame of reference for individual growth.

Duke OHNS has invested in the this journey to ‘train tomorrow's leaders’ with a focus on developing character traits and providing both qualitative and quantitative ways to provide feedback and individualized data that allow for resident and faculty interaction, personal introspection, and the platform for growth. The next steps include broadening our initiative at Duke and with other interested collaborators across the nation. More data and assessment will ensure the development and evolution of STEPP, for all in medicine. Exploration of tools and approaches to developing character strengths in the military should be further pursued.
